# Efficacy of combined use of Suvorexant and Ramelteon in preventing postoperative delirium: a retrospective comparative study

**DOI:** 10.1186/s40780-023-00311-z

**Published:** 2023-12-01

**Authors:** Shoya Ikeuchi, Rei Tanaka, Teiichi Sugiura, Kaori Shinsato, Akane Wakabayashi, Junya Sato, Keiko Suzuki, Michihiro Shino

**Affiliations:** 1 Department of Pharmacy, Shizuoka Cancer Center, Shizuoka, Japan; 2 Drug Eleven Pharmacy Sashiki, Okinawa, Japan; 3 Faculty of Pharmaceutical Sciences, Shonan University of Medical Sciences, Kanagawa, Japan; 4 Division of Hepato-Biliary-Pancreatic Surgery, Shizuoka Cancer Center, Shizuoka Cancer Center, Shizuoka, Japan; 5 Division of Psycho-oncology, Shizuoka Cancer Center, Shizuoka Cancer Center, Shizuoka, Japan

**Keywords:** Postoperative delirium, Suvorexant, Ramelteon, Hypnotic, Preventive efficacy

## Abstract

**Background:**

Suvorexant and ramelteon have been presented as useful for preventing postoperative delirium. Previous studies reported on the comparison with benzodiazepine hypnotics which have been known for the risk for inducing delirium, but the comparison with patients not taking any hypnotics has not been reported yet. Therefore, we assessed the incidence rates for postoperative delirium comparing cancer patients who received preoperative combined administration with suvorexant and ramelteon and those not taking any hypnotics.

**Methods:**

Among 110 cancer patients who underwent surgeries at the Division of Hepato-Biliary-Pancreatic Surgery at the Shizuoka Cancer Center between April 1, 2017 and June 30, 2020, 50 patients who received combined administration with suvorexant and ramelteon from 7 days prior to their surgeries and 60 patients who did not take any hypnotics including suvorexant and ramelteon were classified. They were retrospectively observed during the 7 days from their surgeries onward to compare the cumulative incidence rates for postoperative delirium.

**Results:**

The cumulative incidence rate for postoperative delirium during the 7 days in the combined-administration group was 14.0% (7/50), while that for the no-hypnotic group was 36.7% (22/60), which proved that the incidence rate for the former was significantly low (OR: 0.28, 95%CI: 0.11–0.73, *P* = 0.009).

**Conclusions:**

The present study suggests that the preventive combined administration with suvorexant and ramelteon starting from the preoperative period for cancer patients can be effective in lowering the incidence rate for postoperative delirium.

**Trial registration:**

Retrospectively registered.

## Background

Delirium is defined as a clinical condition developed acutely in which cognitive functional deficiency involving various mental manifestations such as loss of orientation, hallucination and/or delusion, and mood swings. The prevention and treatment for delirium can be critical, because it will increase the risk for such accidents as tumbling or falling, and in the long run, it will affect the life prognosis as the mortality rate for those with delirium can be high [1]. Postoperative delirium, directly derived from an invasive surgery, is one of major clinical issues [2]. The incidence rate for postoperative delirium has been reported as between 6.0 and 31.3%, with such risk factors as advanced age, male and having undergone a highly invasive major surgery [3]. The prevention of delirium essentially includes ensuring a nocturnal sleep and stabilizing a normal pattern of sleep and arousal. However, a single-agent administration with a benzodiazepine hypnotic as a prevention method is not highly recommended, because it can rather increase the risk for the delirium incidence [4–6]. Other drugs which can evoke delirium include opioid, steroid, H_2_ antagonistic agent, anticonvulsant agent, antihistamine agent and anticholinergic agent.

Melatonin and orexin play major roles in stabilizing the normal pattern of sleep and arousal, which is highly associated with the delirium incidence. Melatonin is an endogenous hormone which lowers blood pressure, helps vasodilation, and enhances sleep by lowering core body temperature. Ramelteon works on the melatonin receptor and as a result, induces sleep [7]. Sultan SS et al. reported that their patients who had been provided with the oral administrations of ramelteon on the day of their surgeries indicated significantly lower incidence rate for postoperative delirium than those with no administrations (9.4% vs. 32.7%) [8]. Orexin is a neuropeptide secreted during the day to holds up the arousal state. Suvorexant antagonistically works on the orexin receptor and induces sleep [9]. In the comparative investigation by Hatta et al. for the incidence rates for delirium among the patients on the emergency hospitalization, those who received suvorexant indicated significantly lower incidence rate than the placebo group (0% vs. 17.0%) [10].

Hatta et al. reported in another study on the efficacy of the combined-administration therapy with suvorexant and ramelteon provided before surgeries in preventing postoperative delirium for the patients with various diseases including cancer, heart failure and cerebral stroke [11]. In addition, Booka et al. also reported on the preventive efficacy for postoperative delirium of the combined administration with suvorexant and ramelteon in the study confined to cancer patients [12]. However, there were limitations to their studies in which the patients who didn’t receive the combined administration with suvorexant and ramelteon unexceptionally took the benzodiazepine hypnotics and the patients’ diseases were confined to cancers in the esophageal or the head and neck regions. Therefore, in the present study, we investigated on the patients with cancers treated by surgeries in the hepato-biliary-pancreatic region, which were not included in the previous studies, and compared the incidence rates for the postoperative delirium in a group using combined administration with suvorexant and ramelteon with those in another group using no hypnotics.

## Methods

### Study subjects

Among the cancer patients who underwent surgeries at the Division of Hepato-Biliary-Pancreatic Surgery, the Shizuoka Cancer Center, between April 1, 2017 and June 30, 2020, we classified the patients who were provided with the combined administration with suvorexant and ramelteon within 7 days prior to their surgeries and defined them as the combined-administration group, and those who were not administered any hypnotics including suvorexant and ramelteon within 7 days prior to their surgeries and defined them as the no-hypnotic group. After surveying all the drugs prescribed by the attending physician at the Division of Hepato-Biliary-Pancreatic Surgery, and the drugs prescribed by the physicians at the other departments excluding anesthesiology, as well as the drugs which the patients had brought in from other hospitals, all the applicable patients were extracted from the electronic medical records. The exclusion criteria were those who were treated with any one of anticholinergic drug, antihistamine drug, selective serotonin reuptake inhibitor (SSRI), serotonin-noradrenaline reuptake inhibitor (SNRI), noradrenergic and specific serotonergic antidepressant (NaSSA), tricyclic antidepressant and tetracyclic antidepressant, and those who received a sole regimen with either suvorexant or ramelteon.

### Investigation items

We compared the cumulative incidence rates for delirium between the 2 groups during 7 days and 3 days after the surgeries. The presence or absence of the incidence was retrospectively investigated from the patients’ medical records on the electronic charts entered by doctors, nurses and pharmacists (The researcher: S.I.). Delirium was assessed in accordance with the Intensive Care Delirium Screening Checklist (ICDSC) scale. One point was added for each applicable item in the medical record, and the presence of the incidence was identified when the score was 4 or higher. If there were no applicable items in the medical record, the score was 0. The patients who indicated delirium within 7 days after the surgeries were defined as the delirium group, and those who didn’t were defined as the no-delirium group. We investigated the patient background factors, which may have impacted the incidence of delirium, including age (75 or older/younger than 75), gender, general condition (scoring 0 or lower/1 or higher according to the Eastern Cooperative Oncology Group (ECOG) Performance Status), operative procedure, history of alcohol intake, history of hypertension, history of mental disorder (e.g., anxiety neurosis, depression) and presence or absence of adjuvant remedy (opioid within 7 days prior to the surgery, antidopaminergic drug, H2-receptor antagonist or steroid).

### Statistical analyses

We compared the cumulative incidence rates for delirium during 7 days and 3 days after the surgeries between the combined-administration (with suvorexant and ramelteon) group and the no-hypnotic group using the Fisher’s exact test. The comparison was conducted for the patient background factors including age, gender, general condition, operative procedure, type of cancer, history of alcohol intake, previous disease, and adjuvant remedy in the 2 groups. The Cochrane-Armitage trend test was used for comparing operative procedures, and the Fisher’s exact test was employed for the other factors.

We also employed the univariate logistic regression analysis for comparing the background factors affecting the cumulative delirium incidence during 7 days after the surgeries (i.e., presence or absence of oral administration with suvorexant and ramelteon as a preventive remedy, age, gender, operative procedure, general condition, history of alcohol intake, previous disease, and presence or absence of adjuvant remedy). The surgical procedures were defined in 3 categories: Hepatectomy, pancreatectomy, and others (biliary, overlapping region, etc.). In addition, the background factors indicating *P* < 0.2 were adopted for the multivariate logistic regression analyses. The chi-square analyses were conducted for the adopted background factors based on the degree of freedom and the scale ratio to test the significance of the regression analysis. All these statistical tests were run in the BellCurve for Excel (Social Survey Research Information Co., Ltd.) with the level of statistical significance established at 5%.

### Ethical consideration

The present study was conducted in compliance with the “Ethical Guidance for a Study in Medicine Targeted Humans” and with the approval by the Institutional Review Board of the Shizuoka Cancer Center (approval number: J2020-203-2020-1-3).

## Results

### Comparison of the cumulative incidence rates for the postoperative delirium between the combined-administration (with suvorexant and ramelteon) group and the no-hypnotic group

**Fig. 1:**
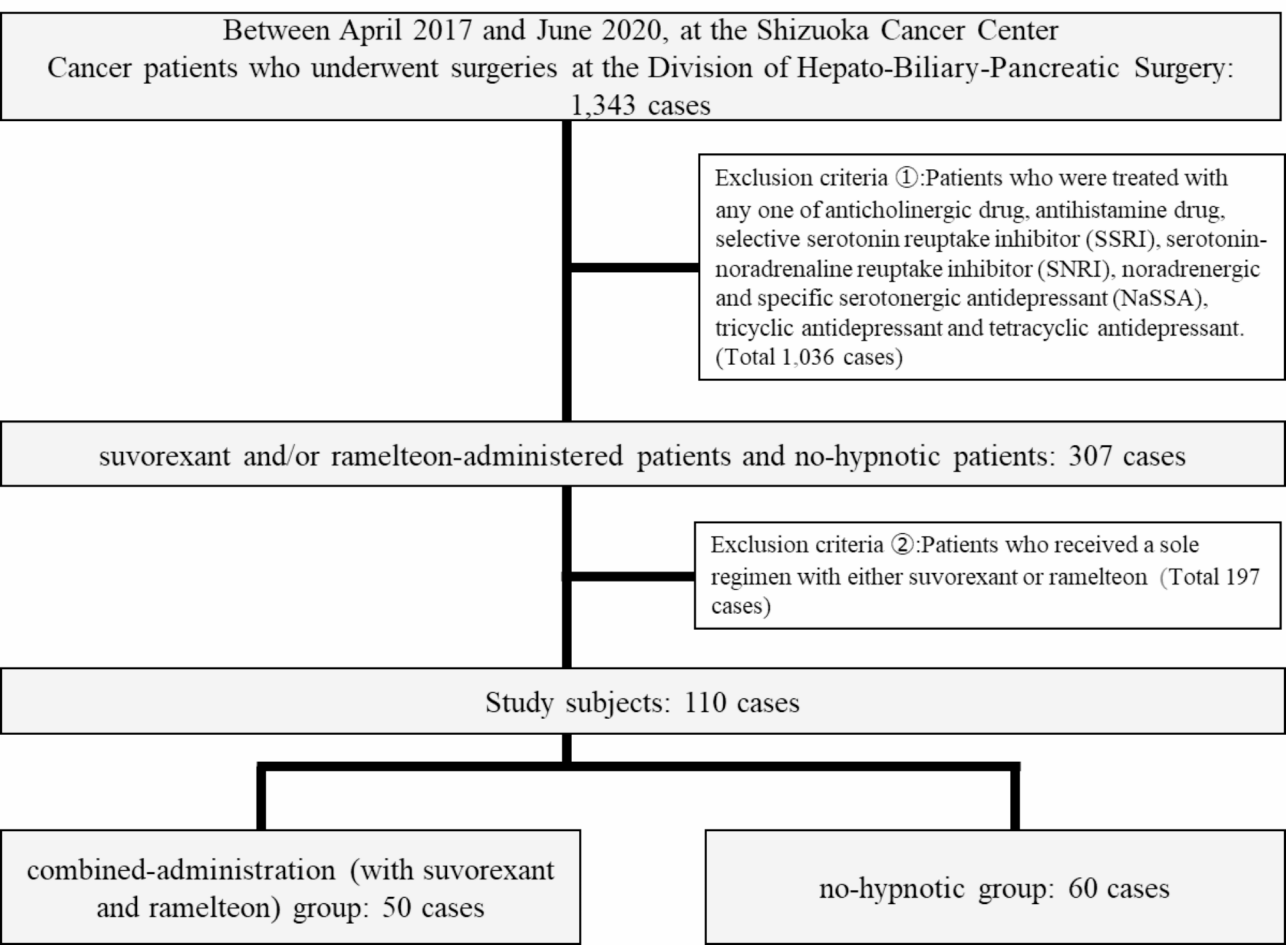
Flow diagram of patient selection

**Fig. 2:**
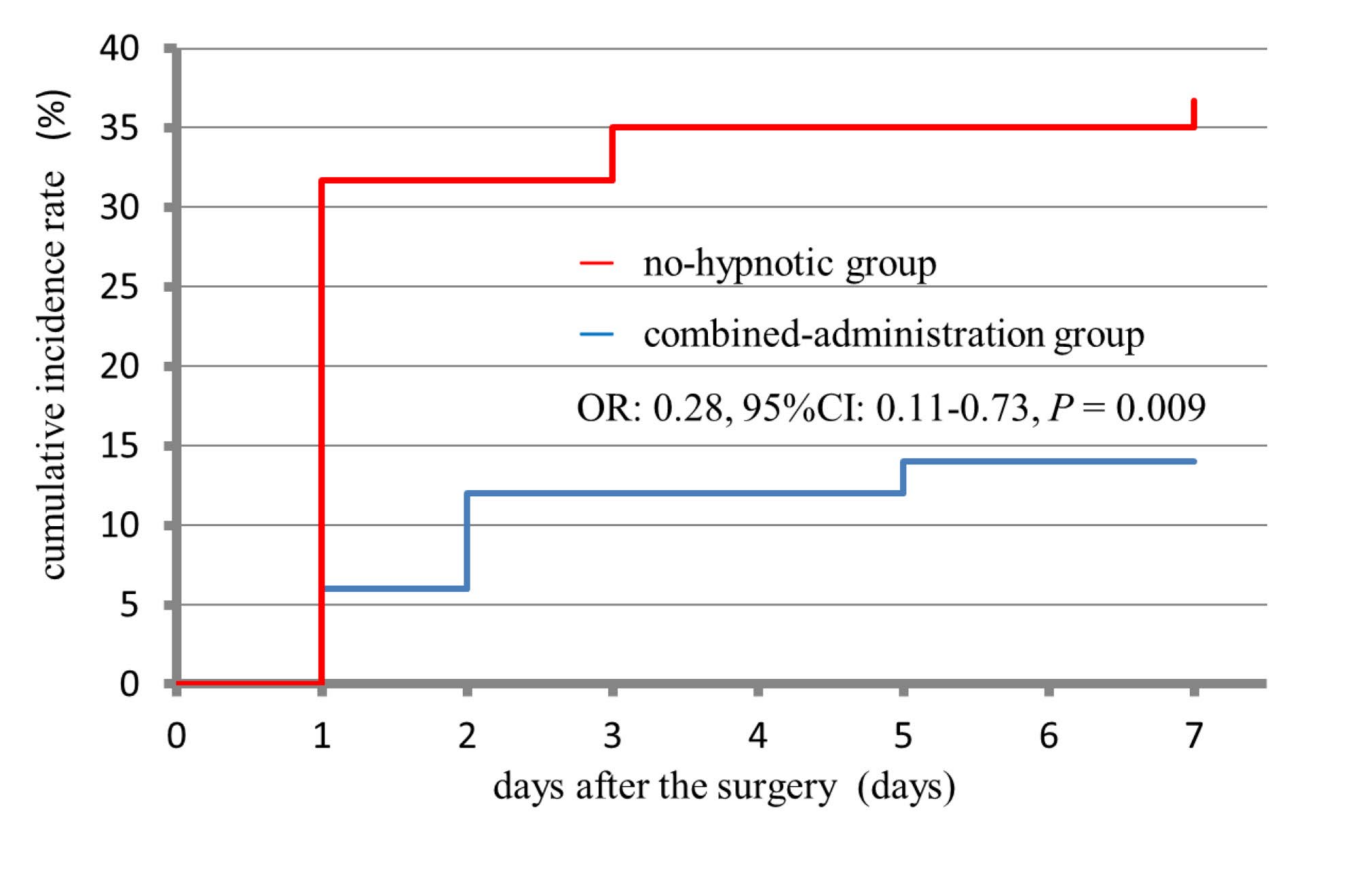
Timing for indication of postoperative delirium

There were 1,343 patients who underwent surgery during the study period. Among them, 1,036 patients who took any one of the following drugs were excluded from the study; anticholinergic drug, antihistamine drug, selective serotonin reuptake inhibitor (SSRI), serotonin-noradrenaline reuptake inhibitor (SNRI), noradrenergic and specific serotonergic antidepressant (NaSSA), tricyclic antidepressant and tetracyclic antidepressant. Also excluded from the study were 197 patients who received a sole regimen with either suvorexant or ramelteon. A total of 110 patients, consisting of 50 in the combined-administration group and 60 in the no-hypnotic group, were ultimately included in the study (Fig. 1). The cumulative incidence rate for the postoperative delirium during 7 days in the combined-administration group was 14.0% (7/50) while that in the no-hypnotic group was 36.7% (22/60), which proved that the incidence rate for the postoperative delirium in the combined-administration group was significantly lower (OR: 0.28, 95%CI: 0.11–0.73, *P* = 0.009) than the latter. And the cumulative incidence rate for the postoperative delirium during 3 days in the combined-administration group was 12.0% (6/50) while that in the no-hypnotic group was 31.7% (19/60), which proved that the incidence rate for the postoperative delirium in the combined-administration group was significantly lower (OR: 0.29, 95%CI: 0.11–0.81, *P* = 0.021) than the latter. The incidence was the most frequently indicated on day 1 and day 2 after the surgeries in the combined-administration group. Day 1 after the surgeries was the most common for the delirium incidence in the no-hypnotic group (Fig. 2).

### Comparison of the patient backgrounds between the combined-administration group and the no-hypnotic group

In comparing the patient background factors, the numbers of the patients aged 75 or older (*P* = 0.031) and having history of mental disorder (*P* = 0.011) were significantly bigger and the number of the male patients was significantly smaller (*P* = 0.021) in the combined-administration group than the no-hypnotic group (Table 1).

**Table 1:** Patient backgrounds

	Combined-admin. group (n = 50)	No-hypnotic group (n = 60)	*P*
Age			
74 or younger	24 (48.0%)	42 (70.0%)	0.031^a^
75 or older	26 (52.0%)	18 (30.0%)	
Gender			
Male	20 (40.0%)	38 (63.3%)	0.021^a^
Female	30 (60.0%)	22 (36.7%)	
Operative procedure			
Hepatectomy	20 (40.0%)	27 (45.0%)	0.373^b^
Pancreatectomy	19 (38.0%)	25 (41.7%)	
Others	11 (22.0%)	8 (13.3%)	
Performance status			
0	32 (64.0%)	36 (60.0%)	0.698^a^
1≤	18 (36.0%)	24 (40.0%)	
History of alcohol intake	31 (62.0%)	41 (68.3%)	0.548^a^
Previous disease			
Hypertension	24 (48.0%)	32 (53.3%)	0.702^a^
Mental disorder	8 (16.0%)	1 (1.7%)	0.011^a^
Adjuvant remedy			
Opioid	0 (0.0%)	2 (3.3%)	0.500^a^
Antidopaminergic drug	0 (0.0%)	1 (1.7%)	1.000^a^
H_2_ receptor antagonist	25 (50%)	29 (48.3%)	1.000^a^
Steroid	0 (0.0%)	3 (5.0%)	0.249^a^

a. Fisher’s exact test

b. Cochrane-Armitage trend test

Logistic regression analyses for the delirium group and the no-delirium group.

In the univariate analyses for the delirium group, which included the patients who indicated delirium within 7 days after the surgeries, and the no-delirium group, which included the patients who didn’t indicate delirium within 7 days after the surgeries, the background factors approved for p < 0.2 were “using the combined remedy with suvorexant and ramelteon,” “aged 75 or older,” “gender (male),” “scoring 0 or 1 in the ECOG performance status,” and “history of hypertension.” As a result of adopting the background factors approved for *P* < 0.2 by the univariate logistic regression analyses and conducting the multivariate logistic analyses with them, the number of the delirium incidences in the patients who received the “combined administration with suvorexant and ramelteon” was significantly small (OR: 0.28, CI: 0.10–0.82, *P* = 0.020) (Table 2). And the significance of the regression analysis in the present logistic analysis was maintained (*P* = 0.002).

**Table 2:** Assessment of suppression factors for postoperative delirium

		Univariate logistic analysis			Multivariate logistic analysis		
		Incidence of delirium within 7 days after the surgery					
		Yes (n = 29)	No (n = 81)	*P*	OR	95%CI	*P*
Combined admin. with suvorexant and ramelteon	Yes (n = 50)	7 (24.10%)	43 (53.1%)	0.009	0.28	0.10–0.82	0.020
	No (n = 60)	22 (75.9%)	38 (46.9%)				
Age	< 75 (n = 66)	14 (48.3%)	52 (64.2%)	0.136	0.54	0.19–1.57	0.260
	≥ 75 (n = 44)	15 (51.7%)	29 (35.8%)				
Gender	Female (n = 52)	9 (31.0%)	43 (53.1%)	0.004	0.48	0.18–1.31	0.156
	Male (n = 58)	20 (69.0%)	38 (46.9%)				
Operative procedure	Hepatectomy (n = 47)	12 (41.4%)	35 (43.2%)	0.864			
	Pancreatectomy (n = 44)	13 (10.3%)	31 (39.5%)	0.618			
	Others (n = 19)	4 (13.8%)	15 (29.6%)	0.658			
Performance status	0 (n = 68)	13 (44.8%)	55 (67.9%)	0.030	0.44	0.17–1.19	0.106
	1≤ (n = 42)	16 (55.1%)	26 (32.1%)				
History of alcohol intake	No (n = 38)	8 (27.6%)	30 (37.0%)	0.353			
	Yes (n = 72)	21 (72.4%)	51 (63.0%)				
History of hypertension	No (n = 54)	9 (31.0%)	45 (55.6%)	0.022	0.51	0.19–1.40	0.194
	Yes (n = 56)	20 (69.0%)	36 (44.4%)				
History of mental disorder	No (n = 101)	28 (96.6%)	73 (90.1%)	0.765			
	Yes (n = 9)	1 (3.4%)	8 (9.9%)				
Adjuvant remedy	Opioid (n = 2)	2 (6.9%)	0 (0%)				
	Antidopaminergic drug (n = 1)	0 (0%)	1 (1.2%)				
	H_2_ receptor antagonist (n = 54)	15 (51.7%)	39 (48.1%)	0.741			
	Steroid (n = 3)	1 (3.4%)	2 (2.5%)	0.786			

## Discussion

In the present study, the comparison of the incidence rates for the postoperative delirium between the combined-administration (with suvorexant and ramelteon) group and the no-hypnotic group as well as the results of the logistic regression analyses in the delirium group and the no-delirium group suggested the efficacy of the combined administration with suvorexant and ramelteon in preventing the postoperative delirium for the cancer patients. These results indicated a similar tendency with the results of the previous study by Hatta et al. investigating the efficacy of the combined administration with suvorexant and ramelteon for the patients with various diseases including heart failure and brain stroke [11]. One of the similarities with the previous studies was the dates of incidence. Days 1 and 2 in the present study for most of the patients who had the postoperative delirium in the combined-administration group and the no-hypnotic group both coincided with the results reported by Booka et al. [12]. Since postoperative delirium is supposed to occur from 10 min to 7 days [13, 14] after surgery, this study examines the cumulative incidence of delirium over a 7-day period. Likewise, a significant difference was also observed in the analysis limited to the period of frequent occurrence of postoperative delirium up to 3 days after the surgeries, suggesting the efficacy of the combination of suvorexant and ramelteon.

According to the report by Booka et al. [12], the incidence rate for the postoperative delirium was 2.4% (1/41) when cancer patients received either the sole remedy with ramelteon or the combined administration with suvorexant and ramelteon. Among these cases, the incidence rate for the postoperative delirium was 0% (0/19) when the patients received the combined administration with suvorexant and ramelteon. The multivariate analyses in the report indicated that the use of ramelteon was the preventive factor for the postoperative delirium, regardless of using suvorexant in combination, in the surgical treatment in the esophageal as well as the head and neck regions (OR: 0.06, CI: 0.0066–0.55). On the other hand, in the present study, the incidence rate for the postoperative delirium could be lowered by using the combined administration with suvorexant and ramelteon. In addition, in the report by Booka et al., the patients who didn’t take either suvorexant or ramelteon unexceptionally received benzodiazepine hypnotics, while those who didn’t receive the combined administration with suvorexant and ramelteon in the present study used no hypnotics at all, which should be noted as a distinctive difference.

Honda et al. reported in their study that the elderly patients and the male ones indicated the risk factors affecting the delirium incidence [3]. Although the multivariate logistic analyses in the present study didn’t indicate any significant differences between the elderly/male patients and the younger/female ones, the results of the univariate logistic analyses reflected a similar trend with the previous report by Honda et al. The reasons why there were not any significant differences between the elderly/male patients and the younger/female ones in the present study include the fact that the number of the study subjects was rather small, and as a result, the statistical power might not have been enough for substantiating the trend. Although the significance of the regression analysis with the present number of factors was maintained at *P* = 0.002, the small number of cases also affected the number of factors for which multivariate logistics analysis could be performed.　Meanwhile, another previous study reported that the incidence rate for the postoperative delirium was higher in female patients than male ones [15]. Therefore, future studies should include study subjects in bigger numbers than the present study in order to gain more precise and extended results to prove how gender and the delirium incidence rate are associated.

There are some limitations in this study. Firstly, the limitations of the present study can be the bias in the patient background factors. In the combined-administration group, as the numbers of the patients aged 75 or older and those with the history of mental disorder were significantly big and the number of male patients was significantly small, age and gender may have affected the delirium incidence rates in each group. In one of the previous studies, Kazmierski et al. reported that the delirium incidence rate after cardiac procedures among the depression patients was significantly high [16]. Among the patient background factors in the present study, the history of mental disorder turned out to be a significantly superior one for the combined-administration group. Secondly, since all the applicable patients were extracted from the electronic medical records by surveying all the drugs prescribed by the attending physician of the Division of Hepato-Biliary-Pancreatic Surgery, and the drugs prescribed by the physicians at other divisions excluding anesthesiology, as well as the drugs which the patients had brought in from other hospitals, some of the cases included unknown intentions for prescribing suvorexant and ramelteon. As the combined remedy with suvorexant and ramelteon was administered for the purpose of improving insomnia in the first place, it is possible to speculate that delirium tended to be induced more often in the combined-administration group than the no-hypnotic group, because insomnia is one of the mental disorders inducing delirium. In addition, it is undeniable that the prescription for suvorexant and ramelteon may have been biased, as the physician who prescribed them could have thought that the patients had high risk for delirium. However, it was controlled well in the present study. If some of the patients in the combined-administration group had similar background factors as the no-hypnotic group, which included the cases where the patients were not elderly and/or didn’t have the history of mental disorder for example, the incidence of delirium in the combined-administration group is well controlled, and the difference in incidence between the two groups may be greater. Thirdly, there were some other factors affecting the delirium incidence rates than those covered in the present study. They include lights in the hospital rooms, nutrition status, and sleep hours for instance. These are the factors which the nursing skills of the ward staff, who are responsible for providing non-drug therapy, matter in most cases and may have possibly affected the delirium incidence rates in the present study [17]. Also noteworthy was that, while longer operation time [18], longer duration of anesthesia [19], and worse postoperative pain control [15, 20] affect the occurrence of postoperative delirium, some of the cases in the present study included no clear descriptions for operation time or endpoints (Numerical Rating Scale, Visual Analogue Scale, etc.). Therefore, comparisons using statistical analysis could not be made. However, the electronic medical records of the patients did not contain any reports on abnormally long operation time, prolonged duration of anesthesia, or increased pains, which suggests that their effects on the surgeries and postoperative management must have been small. Finally, as the investigator (S.I.) in this study was also involved in the analysis itself, this study was not blinded, and the influence of rater bias cannot be denied for concluding that suvorexant-ramelteon is effective in preventing postoperative delirium. It is suggested that if a prospective, blinded study is conducted in the future, in which this bias can be eliminated, the difference between the two groups may be smaller than the present study.

## Conclusion

The combined administration with suvorexant and ramelteon can be suggested as effective for preventing the postoperative delirium for the cancer patients who underwent the hepato-biliary-pancreatic surgeries.

## Data Availability

The dataset supporting the conclusions of this article is included within the article.

## Abbreviations

CI: Confidence interval
ICDSC: Intensive care delirium screening checklist
OR: Odds ratio
PS: Performance status
